# c-MET immunostaining in colorectal carcinoma is associated with local disease recurrence

**DOI:** 10.1186/s12885-015-1662-6

**Published:** 2015-10-12

**Authors:** Jaudah Al-Maghrabi, Eman Emam, Wafaey Gomaa, Moaath Saggaf, Abdelbaset Buhmeida, Mohammad Al-Qahtani, Mahmoud Al-Ahwal

**Affiliations:** 1 Scientific Chair for Colorectal Cancer, King Abdulaziz University, Jeddah, Saudi Arabia; 2 Department of Pathology, Faculty of Medicine, King Abdulaziz University, P.O. Box 80205, Jeddah, 21589 Saudi Arabia; 3 Center of Excellence in Genomic Medicine Research, King Abdulaziz University, Jeddah, Saudi Arabia; 4 Department of Pathology, Faculty of Medicine, Alexandria University, Alexandria, Egypt; 5 Department of Pathology, Faculty of Medicine, Minia University, Al Minia, Egypt; 6 Department of Medicine, Faculty of Medicine, King Abdulaziz University, Jeddah, Saudi Arabia

**Keywords:** Colorectal carcinoma, c-met proto-oncogene, Clinicopathological characteristics, Prognosis

## Abstract

**Background:**

Increased mesenchymal–epithelial transition factor gene (c-MET) expression in several human malignancies is related to increased tumour progression. The aim of the present study is to explore the relationship between immunohistochemical expression of c-MET in colorectal carcinoma (CRC) and the clinicopathological characteristics and follow up data, to compare the expression of c-MET in primary CRC and its metastasis in lymph nodes and to test its validity as independent prognostic factor.

**Methods:**

Hundred and thirty-five archival CRC and nodal metastases samples were collected from King Abdulaziz University Hospital, Saudi Arabia. Tissue microarrays were constructed and immunohistochemistry was done to detected c-MET protein expression. Appropriate statistical analysis was performed.

**Results:**

High c-MET immunostaining was significantly associated with tumour size larger than 5 cm (*p* < 0.003) and in left colon subsite (*p* < 0.05). There was no significant correlation between c-MET protein expression and age, sex, degree of differentiation, tumour invasion, presence of nodal metastasis, lymphovascular invasion, status of surgical resection margin, or presence of distant metastasis. Furthermore, no association between c-MET protein expression and disease free survival. High protein expression of c-MET is associated with the incidence of local disease recurrence (*p* < 0.012).

**Conclusion:**

c-MET is a new promising target that may help in understanding the pathogenesis of CRC, and to be used as independent prognostic biomarker to predict local disease recurrence in CRC. Further molecular *in vitro and in vivo* studies are required to pursue c-MET as potential molecular marker of metastases and test the possibility of its incorporation as a new targeted therapeutic target.

## Background

Colorectal carcinoma (CRC) is among the most common neoplasms affecting the industrialised nations. According to the Saudi Arabian National Cancer Registry, CRC is accounting for 11.3 % of all newly diagnosed cases in year 2009. This cancer ranked first among male population and third among female population [1]. The prognosis of colorectal carcinoma is controlled by variable factors which are classified by the College of American Pathologists [2] into; biological and clinical factors as tumour stage, grade, and molecular factors as genetic markers, DNA contents and markers of angiogenesis and proliferation. According to UICC-TNM and American Joint Committee on Cancer classifications [3] tumour extent, lymph node status, tumour grade, and the assessment of lymphatic and venous invasion are still the most relevant clinicopathological prognostic factors. However, tumours of the same stage can follow significantly different clinical courses, indicating a necessity for the identification of novel prognostic factors, including molecular markers. Therefore, an active search is going on to find powerful new prognostic and predictive molecular markers for identifying high-risk patients who would benefit from individually tailored treatment options [4–6].

One of the most promising biological markers in the pathogenesis of CRC is the proto-oncogenes c-MET (mesenchymal–epithelial transition factor gene) which encodes the tyrosine kinase receptor for hepatocyte growth factor/scatter factor (HGF/SF). Interaction between HGF and c-MET plays a key role in cellular proliferation, survival, migration, and invasion. The HGF/c-MET axis contributes a critical physiological function in embryogenesis, angiogenesis, and wound healing [7, 8]. Under normal conditions c-MET is expressed in vascular cells, lymphatic endothelial cells and hematopoietic cells. Somatic mutation and overexpression of c-MET gene has been described in variety of solid organ tumours including carcinoma of lung, bladder, kidney, thyroid and CRC [7, 9–11]. Over expression of c-MET in such tumours is claimed to be related to increased tumour cell motility, invasiveness, and angiogenesis which may stimulate tumour metastatic ability and contribute to tumour aggressiveness [12–14].

The aim of the present study is to explore the relationship between immunostaining of c-MET in CRC and the clinicopathological characteristics and follow up data, to compare c-MET immunostaining in primary CRC and its metastasis in lymph nodes, and to test its validity as independent prognostic factor.

## Methods

### Patients

The current study involved 135 paraffin blocks retrieved from patient’s materials in the archives of the Pathology Department, King Abdulaziz University, Jeddah, Saudi Arabia covering the period from January 1995 to December 2010. Paraffin blocks were sliced and stained routinely with haematoxylin and eosin to evaluate histopathological characteristics of the tumours as well as for histological grading and staging. Clinical parameters of patients were collected from the patient's medical records and listed in Table 1. The study was approved by the Research Committee of the Biomedical Ethics Unit, Faculty of Medicine, King Abdulaziz University, Jeddah, Saudi Arabia. All patients included in this study gave an informed written consent for utilisation of their material in research and was accepted by Research Committee of the Biomedical Ethics Unit.

**Table 1:** Clinicopathological parameters of cases (*n* = 135)

Parameter		Number (%)
Sex	Male	67 (49.6 %)
	Female	68 (50.4 %)
Grade	Well-differentiated	31 (23 %)
	Moderately-differentiated	85 (63 %)
	Poorly-differentiated	19 (14 %)
Age	<60 years	72 (53.3 %)
	≥60 years	63 (46.7 %)
Tumour location	Right colon	38 (28.1 %)
	Left colon	83 (61.5 %)
	Rectum	14 (10.4 %)
Tumour size	<5 cm	56 (%41.5)
	≥5 cm	79 (58.5 %)
Primary tumour	T1	3 (2.2 %)
	T2	14 (10.4 %)
	T3	108 (80 %)
	T4	10 (7.4 %)
Nodal metastasis	Positive	63 (46.7 %)
	Negative	68 (50.3 %)
	Cannot be assessed	4 (3 %)
Distant metastasis	Positive	40 (29.6 %)
	Negative	95 (70.4 %)
Lymphovascular invasion	Positive	20 (14.8 %)
	Negative	115 (85.2 %)
Margin status	Involved	9 (6.7 %)
	Free	126 (93.3 %)
Local disease recurrence	Recurrence	48 (35.6 %)
	No recurrence	87 (64.4 %)
Survival	Died of disease	26 (19.3 %)
	Alive	89 (65.9 %)
	Not available	20 (14.8 %)

T1: Tumour invades submucosa

T2: Tumour invades muscularis propria

T3: Tumour invades through the muscularis propria into the subserosa or into non-peritonealised pericolic or perirectal tissues

T4: Tumour directly invades other organs or structures, and/or perforates visceral peritoneum

### Tissue microarray construction

A tissue microarray (TMA) was constructed from 135 primary CRC and 49 corresponding lymph nodes showing tumour metastasis. Two cylindrical cores of 1.5 mm in diameter were selected from donor paraffin blocks and arrayed in 6 recipient paraffin blocks using the automated tissue arrayer (MASTER 3D HISTECH). Normal placenta tissue was used as control tissue to help orientation of samples in each TMA block. TMA blocks were sliced into 4-micron meter sections embedded in sialinated slides in order to be stained by immunohistochemical staining.

### Immunohistochemistry of tissue microarray

Immunohistochemical staining of CRC samples were using antibody to protein c-MET was performed by avidin biotin procedure following manufacturer's kit instructions. The antibody used was a monoclonal mouse Anti-Human c-MET (Dako Cytomation Norden A/S, Glostrup, Denmark, dilution 1:100). IHC procedure was carried out using an automatic immunostainer (Ventana Bench Mark XT, Ventana Inc., Tucson, AZ). In each analysis, positive controls were used consisting of CRC samples previously shown to stain with this antibody. Tris-buffered saline in place of the primary antibody was used as a negative control.

### Interpretation of immunohistochemical staining

c-MET protein positivity appeared as yellow to brown staining in tumour cell cytoplasm and/or cell membrane. The immune reaction was evaluated semiquantitavely according to staining intensity taking into account the percentage of cells staining at a given intensity, following the scoring system given by Takeuchi et al. [15] on a scale from 0 to 3, with grade 0: negative, 1: weak, 2: moderate, and 3: intense. When dichotomised for statistical risk assessment, negative (−) and weak (+) staining were defined as low immunostaining, while moderate (++) and intense (+++) staining were included in high immunostaining category.

### Statistical analysis

Differences between two groups of patients on one variable were tested by using Mann Whitney test while between three groups of patients by Kruskal Wallis test was used. Non-parametric chi-square was used to test variance along one variable. Binary logistic regression analysis was used to predict lymph noel metastasis, distant metastasis, surgical resection margins involvement, lymphovascular invasion, and local disease recurrence in relation immunoexpression of c-MET. Estimated odds ratio {exponential (B)}, 95 % confidence interval (CI) for exp(B). The Kaplan-Meier procedure was used to calculate the disease-free survival probabilities and the Log Rank test was used to compare the difference between survivals. Time was calculated from the date of diagnosis to the appearance of disease relapse (or date last seen disease-free). Statistical procedures were performed using SPSS® Release 16.0. Statistical significance was determined at p value of ≤0.05 and was 2-sided.

## Results

### c-MET immunostaining profiles

**Fig. 1:**
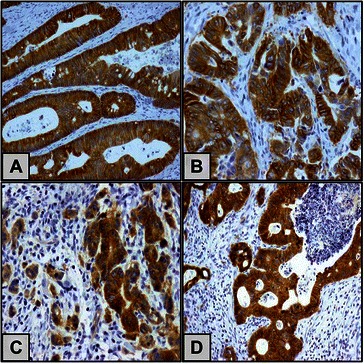
Immunohistochemical labelling of c-MET in CRC and nodal metastasis using anti c-MET antibody. Diaminobenzidine was used as chromogen and haematoxylin as counterstain. A combined cytoplasmic and membranous immunostaining is shown in well differentiated CRC (**a**), moderately differentiated CRC (**b**), poorly differentiated CRC (**c**), and in lymph node metastasis (d). Original magnification used is 200x

In CRC, c-MET protein expression was observed as in tumour cells, but was negative in inflammatory cells, endothelial cells, smooth muscle cells or fibroblasts. c-MET immunostaining positivity was observed as yellow-brown staining observed in the cytoplasm and cell membrane. (Fig. 1) Cases with low c-MET protein expression outnumbered cases showing high c-MET protein expression; low expression: 87 cases (64.4 %) and high expression: 48 cases (35.6 %). There was no difference between c-MET protein expression in primary tumour and in lymph node metastasis (Table 2).

**Table 2:** Categories of c-MET protein immunoexpression

	Primary tumour (*n* = 135)	Nodal Metastasis (*n* = 61)	*p* value
Low expression	87/135 (64.4 %)	46/61 (75.4 %)	0.129^**^
High expression	48/135 (35.6 %)	15/61 (24.6 %)	
*p* value	<0.001^*^	<0.001^*^	

^*^One sample non-parametric chi-square test

^**^Mann–Whitney test

### The relationship between c-MET immunostaining and clinicopathological features of CRCs

There was no significant correlation between c-MET expression and age, sex, degree of differentiation, depth of tumour invasion, tumour stage, presence of nodal metastasis, lymphovascular invasion, status of surgical margins and presence of nodal distant metastasis. On the other hand, high c-MET immunostaining was significantly associated with tumour sizes larger than 5 cm and in left colon location (Table 3).

**Table 3:** Association of c-MET protein immunoexpression with clinicopathological parameters

Parameter	*p* value
Grade	0.079^*^
Sex	0.768^**^
Age	0.566^**^
Tumour location	0.05^*^
Tumour size	0.003^***^
Depth of invasion [2]	0.092^*^
Nodal metastasis	0.682^**^
Lymphovascular invasion	0.287^**^
Margin status	0.886^**^
Local disease recurrence	0.012^***^
Distant metastasis	0.206^**^

^*^Kruskal-Wallis Test

^**^Mann–Whitney test

^***^Signficantly associated with c-MET overexpression

### The relationship between c-MET protein expression and survival outcome

**Fig. 2:**
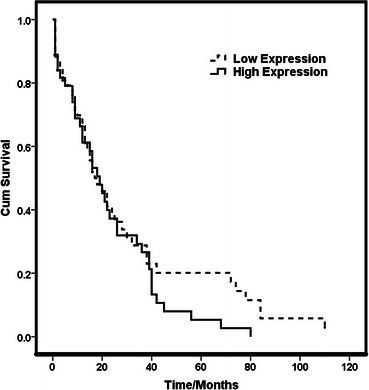
Disease free survival curve (Kaplan-Meier Curve) showing the survival probabilities of low and high immunoexpression groups of c-MET (log-rank = 1.142, *p* = 0.285)

The survival curves for high- and low c-MET groups were assessed by the Kaplan-Meier method and compared by a log-rank test which showed no association of c-MET protein expression with disease-free survival (Log rank = 1.142, and *p* = 0.285) (Fig. 2). Regression analysis for c-MET immunoexpression revealed that high protein expression is associated with the incidence of recurrence and could be an independent predictor of occurrence of recurrence (Table 4).

**Table 4:** Regression analysis for c-MET protein immunoexpression

Variable	Exp (B)	95 % CI for exp (B)	*p* value
Nodal Metastasis	1.163	0.566–2.387	0.681
Distant metastasis	1.682	0.750–3.770	0.207
Surgical resection margins	1.111	0.265–4.657	0. 885
Lymphovascular invasion	1.792	0.608–5.278	0.290
Local disease recurrence	3.322	1.264–8.732	0.015^*^

^*^c-MET overexpression is an independent predictor of the incidence of local disease recurrence

## Discussion

CRC remains the second leading cause of cancer-related death worldwide despite recent advances in adjuvant chemotherapeutic regimens [16]. Recent researches are concerned about better understanding of the pathogenesis and biological characteristics of CRC aiming at development of new targets for therapy. The role of c-MET signalling has not been fully elucidated in CRC. c-MET is aberrantly activated in many human cancers through diverse mechanisms, including point mutations, gene amplification, transcriptional up-regulation, or ligand autocrine loops.

The present study showed that c-MET protein is concomitantly overexpressed in both primary tumours and in nodal metastasis which is similar other studies findings [17, 18]. The current study demonstrates that high protein expression of c-MET protein using immunohistochemical technique was demonstrated to be significantly associated with large tumour size, and left side location of the tumour. However, other studies Zeng et al., [19] and Liu et al., [20] did not find any correlation between c-MET and tumour site or size [19, 20]. We did not find any significant correlation between c-MET protein overexpression and tumour stage, distant metastasis, or lymph node positivity contrasting results of previous studies [15, 19, 20]. In Takeuchi et al. study, they found high c-MET immunostaining was associated with advanced stages [15]. Although the same scoring system was used, their sample size was relatively smaller than in the current study. Zeng et al. observed C-MET gene amplification in advanced primary tumour stages. Additionally, Liu et al. showed c-MET mRNA and protein overexpression in primary CRC with high stage and positive nodal metastasis [20]. The discrepancy from the present study may be due to smaller number of tumours. In the current study, there was no association between c-MET protein overexpression and disease free survival which is similar to previous report [18]. In another studies, c-MET immunostaining was found to be associated with overall survival [17, 21]. This discrepancy may be attributed to small sample size and using a different immunostaining scoring system.

Significant statistical correlation was identified between c-MET protein overexpression and tumour recurrence. This result is comparable to those of Saigusa et al. [9] that investigated the relation between c-MET protein expression and distant recurrence following preoperative chemotherapy in rectal carcinoma and the effect of c-MET inhibition on tumour cell growth after radiotherapy. They demonstrated that c-MET protein overexpression is related to tumour recurrence and is associated with worse prognosis and suggested that inhibition of c-MET is a potential new strategy for reduction of distant recurrence of rectal carcinoma after preoperative chemotherapy.

In addition to association of c-MET activation with tumour progression, previous studies demonstrated that the HGF/c-MET signalling pathway plays a role in angiogenesis and lymphangiogenesis by promoting the growth of endothelial cells, increasing the expression of pro-angiogenic mediators, such as vascular endothelial growth factor, and suppressing the activity of thrombospondin 1-a negative regulator of angiogenesis. Considering the relevant role of c-MET in angiogenesis, activated c-MET is considered as adjuvant pro-metastatic gene for many tumour types. Novel therapeutic strategies, which focus on the simultaneous blockade of such proto-oncogene, have been recently proposed to improve the treatment outcome [22–24].

The limitation of the current study includes including missing some follow-up data, short survival time in a number of patients.

## Conclusions

In summary, in the present study the association of high c-MET immunostaining with larger tumours supports involvement of c-MET in tumour progression. On the other hand, c-MET is a promising target that may help in understanding the pathogenesis of CRC, and to be used as independent prognostic biomarker to predict local disease recurrence in CRC. Further molecular *in vitro and in vivo* studies are required to pursue c-MET as potential molecular marker of metastases and test the possibility of its incorporation as a new targeted therapeutic target.

## Abbreviations

CRC: Colorectal carcinoma
HGF: Hepatocyte growth factor
UICC: Union for International Cancer Control
TNM: Tumour, Node Metastasis
TMA: Tissue microarray
